# Antibiotic treatment In patients with chronic low back pain and Modic changes (the AIM study): study protocol for a randomised controlled trial

**DOI:** 10.1186/s13063-017-2306-8

**Published:** 2017-12-15

**Authors:** Kjersti Storheim, Ansgar Espeland, Lars Grøvle, Jan Sture Skouen, Jörg Aßmus, Audny Anke, Anne Froholdt, Linda M. Pedersen, Anne Julsrud Haugen, Terese Fors, Elina Schistad, Olav Lutro, Gunn Hege Marchand, Thomas Kadar, Nils Vetti, Sigrun Randen, Øystein Petter Nygaard, Jens Ivar Brox, Margreth Grotle, John-Anker Zwart

**Affiliations:** 1 Research and Communication Unit for Musculoskeletal Health (FORMI), Oslo University Hospital Ullevål, Pb 4950, Nydalen, 0424 Oslo, Norway; 2 Department of Radiology, Haukeland University Hospital, Jonas Liesvei 65, 5021 Bergen, Norway; 3 Department of Clinical Medicine, University of Bergen, Pb 7804, 5020 Bergen, Norway; 4 Department of Rheumatology, Østfold Hospital Trust, Pb 300, 1714 Grålum, Norway; 5 Department of Physical Medicine and Rehabilitation, Haukeland University Hospital, Jonas Liesvei 65, 5021 Bergen, Norway; 6 Department of Global Public Health and Primary Care, Physiotherapy Research Group, University of Bergen, Bergen, Norway; 7 Competence Center for Clinical Research, Haukeland University Hospital, Jonas Liesvei 65, 5021 Bergen, Norway; 8 Department of Rehabilitation, University Hospital of North Norway, Tromsø, Norway; 9 Faculty of Health Sciences, Department of Clinical Medicine, UiT The Arctic University of Norway, Tromsø, Norway; 10 Department of Neurology, Rheumatology and Habilitation (NRH), Drammen Hospital, Vestre Viken Hospital Trust, Pb 800, 3004 Drammen, Norway; 11 Department of Physical Medicine and Rehabilitation, Oslo University Hospital, Ulleval, Pb 4950, Nydalen, 0424 Oslo, Norway; 12 Medical Department, Haukeland University Hospital, Jonas Liesvei 65, 5021 Bergen, Norway; 13 Medical Department, Stavanger University Hospital, Pb 8100, 4068 Stavanger, Norway; 14 Department of Physical Medicine and Rehabilitation, St. Olavs Hospital, Trondheim, Norway; 15 Department of Neuromedicine and Movement Science, Norwegian University of Science and Technology (NTNU), Trondheim, Norway; 16 Department of Neurosurgery, St. Olavs University Hospital, Trondheim, Norway; 17 National Advisory Unit on Spinal Surgery, Norwegian University of Science and Technology (NTNU), Trondheim, Norway; 18 Department of Neuroscience, Norwegian University of Science and Technology (NTNU), Trondheim, Norway; 19 Faculty of Medicine, University of Oslo, Oslo, Norway; 20 Oslo and Akershus University College of Applied Sciences, Faculty of Health Sciences, Department of Physiotherapy, Oslo, Norway

**Keywords:** Chronic low back pain, Antibiotics, Modic change, Randomised controlled trial

## Abstract

**Background:**

A previous randomised controlled trial (RCT) of patients with chronic low back pain (LBP) and vertebral bone marrow (Modic) changes (MCs) on magnetic resonance imaging (MRI), reported that a 3-month, high-dose course of antibiotics had a better effect than placebo at 12 months’ follow-up. The present study examines the effects of antibiotic treatment in chronic LBP patients with MCs at the level of a lumbar disc herniation, similar to the previous study. It also aims to assess the cost-effectiveness of the treatment, refine the MRI assessment of MCs, and further evaluate the impact of the treatment and the pathogenesis of MCs by studying genetic variability and the gene and protein expression of inflammatory biomarkers.

**Methods/design:**

A double-blinded RCT is conducted at six hospitals in Norway, comparing orally administered amoxicillin 750 mg, or placebo three times a day, over a period of 100 days in patients with chronic LBP and type I or II MCs at the level of a MRI-confirmed lumbar disc herniation within the preceding 2 years. The inclusion will be stopped when at least 80 patients are included in each of the two MC type groups. In each MC type group, the study is designed to detect (*β* = 0.1, *α* = 0.05) a mean difference of 4 (standard deviation 5) in the Roland Morris Disability Questionnaire score between the two treatment groups (amoxicillin or placebo) at 1-year follow-up. The study includes cost-effectiveness measures. Blood samples are assessed for security measures and for possible inflammatory mediators and biomarkers at different time points. MCs are evaluated on MRI at baseline and after 12 months. A blinded intention-to-treat analysis of treatment effects will be performed in the total sample and in each MC type group.

**Discussion:**

To ensure the appropriate use of antibiotic treatment, its effect in chronic LBP patients with MCs should be re-assessed. This study will investigate the effects and cost-effectiveness of amoxicillin in patients with chronic LBP and MCs at the level of a disc herniation. The study may also help to refine imaging and characterise the biomarkers of MCs.

**Trial registration:**

ClinicalTrials.gov, ID: NCT02323412. Registered on 21 November 2014.

**Electronic supplementary material:**

The online version of this article (doi:10.1186/s13063-017-2306-8) contains supplementary material, which is available to authorized users.

## Background

Low back pain (LBP) is considered the single leading cause for disability worldwide and affects all age groups from adolescents to the elderly, causing activity limitation and work absence with subsequently an economic burden on individuals, families, communities, industry, health services and governments [1]. It is generally recognised that the etiology of LBP is multifactorial, but despite the increase in research over the last 50 years, there is a lack of knowledge, particularly regarding the interaction between environmental, biological and genetic factors. The use of magnetic resonance imaging (MRI) in the assessment of LBP has increased during the last decade, but in about 80–90% of LBP patients imaging reveals no findings with clear relevance to treatment choice [2]. In recent years Modic changes (MCs) have gained attention; these are MRI signal changes in the vertebral bone marrow extending from the endplate, and are defined into types I (oedema type), II (fatty type) and III (sclerotic type) based on their T1 and T2 signals [3]. MCs have an inconsistent relationship to LBP [4–9] and an unclear pathogenesis, which is hypothesised to be mechanical, autoimmune, vascular, inflammatory and/or infectious [10–14].

Systematic reviews reveal that existing treatments for non-specific LBP have only a small-to-moderate effect [15–17]. One study assessed antibiotic treatment as a new treatment for selected patients with persistent LBP and type I MCs at the level of a previously herniated intervertebral disc [18]. They hypothesised that a low-grade infection of the disc by the anaerobic *Proprionibacterium acnes* bacteria (*P. acnes*) resulted in adjacent bone oedema (type I MCs) and chronic disabling LBP. The hypothesis was based on the investigation of bacterial growth in herniated disc material removed by surgery [19–21], suggesting that those who developed type I MCs were more likely to have infection with *P. acnes*. In a randomised controlled trial (RCT), 100 days of antibiotic treatment with amoxicillin clavulanate tablets was more effective than placebo in all primary and secondary outcomes at 12 months’ follow-up [18].

The effect of antibiotic treatment in chronic LBP patients with MCs should be re-assessed for many reasons. There are controversies regarding this trial which call for further research [22–24]. Long-term antibiotic treatment has side effects and may cause antimicrobial resistance and should not be used in a large sub-group of LBP patients based on a single RCT (16 to 62% of LBP patients have MCs [25, 26]). Furthermore, the rationale for the treatment is uncertain. The neovascularisation associated with disc herniation is thought to allow *P. acnes* to enter the disc during ‘normal’ bacteraemia [19, 21], but there is conflicting data on the existence of *P. acnes* in discs with or without herniation [23, 24, 27, 28]. The presence of *P. acnes* in the discs of patients undergoing spinal surgery ranges from 0 to 86% [24] and it can also not be excluded that the bacteria found in nuclear material from discs that have been operated on may be due to intraoperative contamination rather than infection [23, 24]. Additionally, the previous trial only included patients with type I MCs [18]. Until now, no study has examined whether antibiotic treatment is effective for patients with type II MCs. Furthermore, the 0.2-Tesla MRI used in the previous study may have shown substantially more type I MCs and fewer type II MCs compared with a 1.5-Tesla MRI which is commonly used in clinical practice [29]. One may also question the relevance of distinguishing between type I and type II MCs since they may represent different stages of a common process [3, 30].

The present article details the protocol related to the main purpose of the current study, which is to examine the effects of antibiotic treatment in patients with chronic LBP and type I or type II MCs at the level of a lumbar disc herniation. The study also aims to assess the cost-effectiveness of the treatment, refine the MRI assessment of MCs, and further evaluate the impact of the treatment and the pathogenesis of MCs by studying genetic variability and the gene and protein expression of biomarkers. These parts of the study are only briefly summarised here.

## Methods/design

The study is a multicentre, parallel-group, double-blind, randomised, placebo-controlled, phase-III trial. Pre-specified objectives and hypotheses are listed in Table 1. The Regional Committees for Medical Research Ethics in Norway (REC South East, reference number 2014-2029) and the Norwegian Medicines Agency (SLV, reference number 14/01368-11, EudraCT Number: 2013-004505-14) have approved the protocol, which is registered at ClinicalTrials.gov under the identifier: NCT02323412. The project adheres to the Helsinki Declaration, the ICH-GCP (Good Clinical Practice) guidelines and the Consolidated Standards of Reporting Trials (CONSORT) guidelines for the transparent reporting of trials. It is monitored by the Department of Clinical Research Support, Oslo University Hospital.

**Table 1:** Objectives and hypotheses of the AIM study

Objectives	Hypotheses
Main objective	Main hypothesis
To evaluate the effect of amoxicillin versus placebo on disease-specific disability evaluated by the RMDQ at 1-year follow-up in patients with chronic LBP and MCs type I or II at the level of a previously herniated disc	Patients with MCs type I or II at baseline in the antibiotic treatment group report a significantly lower RMDQ score at 1-year follow-up than patients in the placebo group (hypothesis A)
Secondary objective (SO 1)	Secondary hypotheses
To evaluate the effect of amoxicillin versus placebo on RMDQ at 1-year follow-up separately in patients with type I and type II MCs, respectively	Patients with MCs type I at baseline in the antibiotic treatment group report a significantly lower RMDQ score at 1-year follow-up than patients in the placebo group (hypothesis B) Patients with MCs type II at baseline in the antibiotic treatment group report a significantly lower RMDQ score at 1-year follow-up than patients in the placebo group (hypothesis C)
Key supportive (KSOs) and exploratory objectives	Further hypotheses
To evaluate the effect of amoxicillin versus placebo on ODI at 1-year follow-up in the whole cohort of included patients (KSO 2)	Patients with MCs type I or II at baseline in the antibiotic treatment group report a significantly lower ODI score at 1-year follow-up than patients in the placebo group (hypothesis D)
To evaluate the effect of amoxicillin versus placebo on LBP intensity at 1-year follow-up in the whole cohort of included patients (KSO 3)	Patients with MCs type I or II at baseline in the antibiotic treatment group report a significantly lower LBP intensity NRS score at 1-year follow-up than patients in the placebo group (hypothesis E)
To evaluate whether the short tau inversion recovery (STIR) signal (intensity and extent) of MCs on baseline MRI predicts RMDQ score at 1-year follow-up (KSO 4)	In the antibiotic treatment group, high signal from MCs on STIR at baseline MRI predicts a lower RMDQ score at 1-year follow-up (hypothesis F)
To assess whether change in STIR signal (intensity and extent) of MCs from baseline to 1-year follow-up is related to RMDQ score at 1-year follow-up (KSO 5)	Reduced signal from MCs on STIR from baseline to 1-year follow-up MRI is associated with a lower RMDQ score at 1-year follow-up (hypothesis G)
To evaluate the effect of amoxicillin versus placebo on health-related quality of life (the EQ-5D) at 1-year follow-up in the whole cohort of included patients (KSO 6)	Patients with MCs type I or II at baseline in the antibiotic treatment group report significantly better quality of life (EQ-5D) at 1-year follow-up than patients in the placebo group (hypothesis H)
To evaluate cost-effectiveness of amoxicillin versus placebo at 1-year follow-up in the whole cohort of included patients	
To evaluate the difference in incidence of AEs and SAEs between the two intervention groups from inclusion to 1-year follow-up in the whole cohort of included patients	
To investigate the effect of amoxicillin on epigenetic patterns, longitudinal gene and protein expression, genetic variation, from baseline to post treatment (100 days after start of treatment) and from baseline to 1 year (12 months’) follow-up in patients with MCs type I or II, and to evaluate correlations with clinical data	
To investigate the effect of amoxicillin on bowel flora, resistant bacteria and resistance genes	
To evaluate whether positive pain provocation tests at baseline predicts RMDQ score at 1-year (12 months’) follow-up	
Secondary clinical outcomes not specified above will be used to explore hypotheses regarding clinical effects post treatment and 1 year after start of treatment in the whole cohort and separately in patients with type I and type II MCs	

*RMDQ* Roland Morris Disability Questionnaire, *MCs* Modic changes, *KSO* key supportive objectives, *ODI* Oswestry Disability Index, *LBP* Low back pain, *NRS* Numerical Rating Scale, *STIR* short tau inversion recovery, *MRI* magnetic resonance imaging, *AE* adverse event, *SAE* serious adverse event

### Study population and recruitment

Patients with chronic LBP referred to outpatient clinics at participating hospitals (Oslo University Hospital, Ullevål; Haukeland University Hospital; Bergen; St. Olavs Hospital, Trondheim; University Hospital of North Norway, Tromsø and Østfold Hospital Trust, Moss, Drammen Hospital) are screened for eligibility. Both conservatively treated patients and patients operated on for disc herniation more than 12 months prior to inclusion are eligible. In addition, patients registered in the Norwegian Registry for Spine Surgery who have been operated on for disc herniation and report severe LBP at 1-year follow-up in the registry, are eligible. A total of at least 160 patients will be included and randomised (see ‘Sample size’ section below).

To be included in the trial all participants must satisfy all of the following inclusion criteria:

- Age between 18 and 65 years
- LBP of more than 6 months duration in the area below the 12th rib and above the gluteal folds with a Numerical Rating Scale (NRS) pain intensity score of ≥ 5 (mean of three 0–10 NRSs: current LBP, the worst LBP within the last 2 weeks, and the usual/mean LBP within the last 2 weeks)
- MRI-confirmed lumbar disc herniation within the preceding 2 years
- Type I and/or type II MC in the vertebral body marrow at the same level as the previously herniated disc. For patients with previous surgery for disc herniation, the MC has to be located at level that has been operated on
- Written informed consent

The exclusion criteria are as follows:

- Allergy to penicillin or cephalosporins
- Allergy/hypersensitivity to any of the excipients of the study drug
- Current pregnancy or lactation
- Kidney (creatinine) or hepatic (ALAT/ASAT) laboratory values above the normal range
- Phenylketonuria (Følling’s disease)
- Mononucleosis or leukaemia
- Any specific diagnosis that may explain the patient’s low back symptoms (e.g., tumour, fracture, spondyloarthritis, infection, spinal stenosis)
- Previous low back surgery (L1–S1) for reasons other than disc herniation (e.g., fusion, decompression, disc prosthesis)
- Surgery for disc herniation within the last 12 months
- Previous surgery for disc herniation, but MC located at level(s) that has/have not been operated on only
- Reservation about the intake of gelatine (the capsules used to encapsulate the study medicine contains gelatine, which, among other things, is produced using ingredients derived from pigs)
- Regular use of glucocorticoids
- Regular use of opioids with the exception of codeine and tramadol
- Not understanding Norwegian language
- Unlikely to adhere to treatment and/or complete follow-up (e.g., serious ongoing psychiatric disease, drug abuse, plans to move)
- Antibiotic treatment within the month preceding the start of treatment
- Contraindications to MRI (e.g., cardiac pacemaker electrodes, metal implant in the eye or brain, claustrophobia)
- Unwilling to participate

The recruiting clinician screens eligible patients for inclusion and exclusion criteria and requests a baseline study MRI to confirm and characterise MCs seen on the clinical MRI available at screening. The baseline MRI is performed at the local study site and is independently evaluated by two study radiologists, who also re-assess the finding of disc herniation on MRI taken the last 2 years and resolve disagreements relevant to inclusion in consensus. All study sites use the same MRI protocol and the same type of 1.5-Tesla MRI scanner. The radiologists allocate patients to a *MCs type I group* if baseline MRI shows type I MCs (primary or secondary type I at the superior and/or inferior endplate) at a level with disc herniation in the last 2 years. They allocate patients to a *MCs type II group* if baseline MRI shows primary or secondary type II MCs – but not type I MCs – at a level with disc herniation in the last 2 years. Disc herniation is defined as displacement of disc material that is focal, i.e., involves < 25% of the disc circumference (whereas bulges involve > 25% and usually extend < 3 mm beyond the edges of the ring apophyses [31]. Primary (most extensive) and secondary MC types are defined as type I (hypointense T1 signal and hyperintense T2 signal), type II (hyperintense T1 signal and iso/hyperintense T2 signal) and type III (hypointense T1 and T2 signal) [3, 32]. MCs in the vertebral corners with a diameter ≤ 5 mm and MCs with craniocaudal size < 10% of the vertebral height do not qualify for inclusion in the RCT. Treatment starts within 6 weeks after screening and within 4 weeks after the baseline study MRI.

### Randomisation and blinding

Eligible patients are randomised to one of two treatments, amoxicillin or placebo. In order to evaluate treatment effect in all patients as well as separately in each MC type group (I/II), inclusion is planned to continue until each MC type group contains at least 80 patients (see ‘Sample size’ section below). Stratifying for previous disc surgery will ensure a balanced distribution of this potential source of infection between treatment groups (amoxicillin/placebo). A statistician who is not involved in the trial used STATA 13 (StataCorp LP, College Station, TX, USA) to generate the randomisation lists stratified by MC type group (I/II) and previous disc herniation surgery (yes/no) with a 1:1:1:1 allocation. This ensures that balanced numbers of patients receiving antibiotics or placebo within each of the four resulting strata. The patient allocation is performed using the Viedoc™ application. We buy original amoxicillin tablets (Amimox) directly from the manufacturer (Ameda). Kragerø Tablettproduksjon AS, Kragerø, Norway encapsulates the amoxicillin, and produces and encapsulates the placebo. The original amoxicillin tablets are put into a gelatine capsule (Capsugel DB-caps AAel Swedish orange) consisting of two parts put together and firmly tightened to make them as difficult to open as possible. Placebo is put into identical capsules, thus ensuring that both types of capsule have the same look, feel and taste. The study medicine is then filled into identical containers and packed in two different kits, one with capsules containing amoxicillin and the other with capsules containing placebo. Finally, each container is labelled with a study number according to the randomisation list. At inclusion, the patient receives medicine marked only with the study number; the appearance of the containers, labelling, and the capsules themselves are thus identical for both treatment groups. Three containers, each filled with 100 capsules, are handed out to the patient at the start of intervention. Treatment allocation is concealed for all people involved in the trial. A list linking the study number to the active substance is kept at Kragerø Tablettproduksjon AS. The list will be retrieved only at the end of the study when all the data have been collected and entered into the statistical programme and the analysis of the effect of the treatment on the main outcome (see below) is completed.

### Treatment interventions

The previous study used amoxicillin-clavulanate 1500/375 mg/day and 3000/750 mg/day (500/125 mg and 1000/250 mg three times a day) for the low- and high-dose study arm, respectively [18]. The study indicated a non-significant trend towards better outcomes in the high-dose arm at 1-year follow-up, but it was not designed to assess dose response. In Norway, amoxicillin is only available without clavulanate. European culture studies of *P. acnes* do not suggest beta-lactamase [33], and adding clavulanate might increase the risk of side effects. Hence, in the present study, patients are randomised to receive encapsulated amoxicillin tablets 750 mg or placebo three times a day, for 100 days.

Patients are permitted to continue with their usual LBP therapy (e.g., exercises, physiotherapy, analgesics), but are encouraged to limit any intake of non-steroidal anti-inflammatory drugs (NSAIDs) (like ibuprofen or naproxen), which may be a significant co-intervention. Patients treated with drugs potentially causing adverse interaction with amoxicillin (e.g., allopurinol, digoxin, anticoagulants or methotrexate) can be included, but alternatives to these drugs are considered during the intervention period. All adjunctive therapy is registered.

### Data collection

**Fig. 1:**
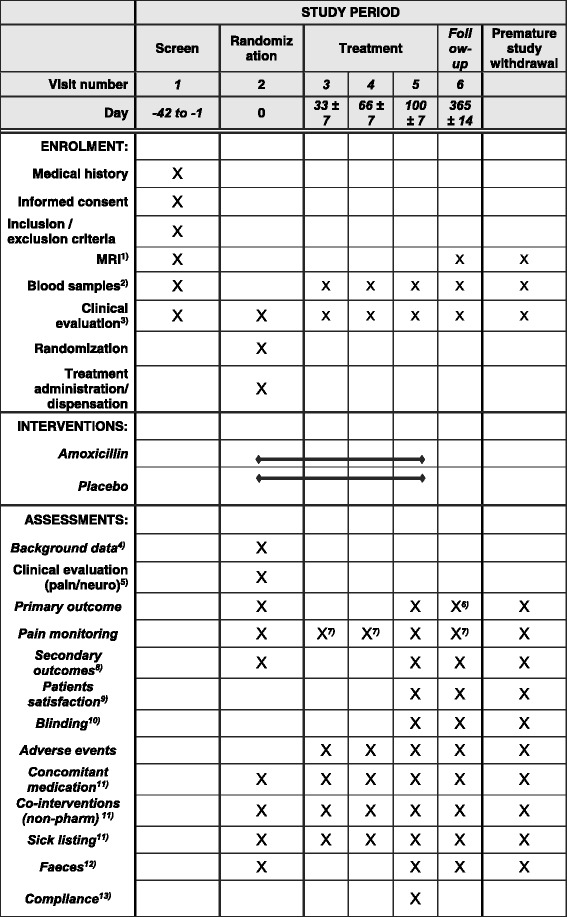
Standard Protocol Items: Recommendations for Interventional Trials (SPIRIT) flow chart. Magnetic resonance imaging (MRI). (1) Baseline MRI according to the study protocol can be a maximum of 4 weeks old when treatment starts. A follow-up MRI is taken between 12 and 13 months after treatment start (i.e., 12 to 14 months after baseline MRI); (2) For safety: haematological parameters (leucocytes, thrombocytes, eosinophils, haemoglobin (Hb) and hematocrit (Ht)) and measures of kidney (creatinine) and liver function (ASAT/ALAT), every month or more frequently if clinically indicated. For scientific purposes: glucose, white cell counts and C-reactive protein (CRP) for further safety monitoring and evaluation of inflammatory mechanisms and genetics/epigenetics; (3) Blood pressure, pulse, auscultation of heart and lungs (safety); (4) Age, gender, Body Mass Index (BMI), ethnicity, marital status, educational level, work status, physical work load, leisure time activity, smoking habits, subjective health complaints, emotional distress, fear-avoidance beliefs, low back pain (LBP) history/duration (including previous treatment, e.g., surgery for disc herniation, physiotherapy, chiropractic), expectations about treatment effect, pain drawing; (5) Pain provocation tests (springing test, active flexion/extension of the lumbar spine) and neurological tests (muscle strength, toe-heel walking, sensibility, reflexes, straight-leg raising, i.e., Lasegue test/reverse Lasegue test); (6) Roland Morris Disability Questionnaire, also collected 6 and 9 months after start of treatment; (7) Pain monitoring (LBP intensity) weekly during treatment period, and at 6 and 9 months after start of treatment; (8) Oswestry Disability Index, leg pain, hours with low back pain during the last 4 weeks, symptom-specific well-being, health-related quality of life, sick leave, short tau inversion recovery (STIR) signal of Modic changes; (9) Patient’s satisfaction with treatment (5-point Likert scale) and global perceived effect (7-point Likert scale); (10) Patients are asked to report which study medicine they think they received (antibiotics/placebo/unsure); (11) Co-interventions (concomitant medication and non-pharmacological treatments) and sick-listing is monitored monthly also during the follow-up period (100 to 365 days) for health-economical calculations; (12) Embraces patients from two participating hospitals. At day 0, faeces are collected before the first tablet is administered; (13) Containers and capsules delivered to local ‘*Sykehusapotek*’ at each participating hospital for return capsule count, registering of accountability in electronic systems and destruction of the returned study drug

Data are collected at baseline, during the 100 days of treatment and during the 9 months after treatment ends (see Fig. 1 for the Standard Protocol Items: Recommendations for Interventional Trials (SPIRIT) flow chart). Hence, patients are embedded in the trial for 1 year after inclusion. Data are collected regardless of participants’ compliance with the study protocol.

Background data are collected at baseline only and include patient age, gender, Body Mass Index (BMI), ethnicity, marital status, educational level, work status, physical work load, leisure time activity, smoking habits, subjective health complaints [34], emotional distress (Hopkins Symptom Checklist-25 (HSCL-25)) [35], fear-avoidance beliefs (Fear Avoidance Beliefs Questionnaire (FABQ) work/physical activity) [36], LBP history/duration (including previous treatment, e.g., surgery for disc herniation, physiotherapy, chiropractic), expectations about treatment effect, and drawing of pain distribution (localised versus widespread pain) [37, 38]. In addition, pain provocation tests (springing test, active flexion/extension of the lumbar spine) and neurological tests (muscle strength, toe-heel walking, sensibility, reflexes, straight-leg raising, i.e., Lasegue test/reverse Lasegue test) are structured and registered by a clinician in a Case Report Form (CRF) at baseline.

### Primary outcome

The primary outcome measure is pain and disability measured by the Norwegian version of the Roland and Morris Disability Questionnaire (RMDQ) [39, 40]. The RMDQ scale ranges from 0 to 24; higher scores indicate worse disability. The RMDQ was also used as the primary outcome in the previous trial [18].

### Secondary outcomes

Secondary clinical outcome measures are pain-related disability assessed by the Oswestry Disability Index (ODI) version 2.0 [41], LBP intensity (mean 0–10 NRS score for current LBP, worst LBP within the last 2 weeks, and usual/mean LBP within the last 2 weeks), leg pain last week (0–10 NRS), hours with LBP during the last 4 weeks (range 0–448 h) [18], symptom-specific well-being (5-point Likert scale) [42, 43], health-related quality of life (EuroQoL-5D version 2.0) [44], self-reported days of sick leave, co-interventions (other pharmacological (ATC-coded) and non-pharmacological treatments), patient’s satisfaction with treatment (5-point Likert scale) and global perceived effect (7-point Likert scale). A further secondary outcome measure is the short tau inversion recovery (STIR) signal of MCs.

### Adverse events and safety

Details of adverse events are assessed by the clinician and are MedDRA-coded in a CRF during the treatment period (monthly), post treatment and at 1-year follow-up. Haematological parameters, including measures of kidney and liver function, are assessed monthly during the treatment period, together with a short clinical evaluation to monitor side effects (not outcome measures).

### Compliance and blinding assessment

Treatment compliance is assessed by capsule counts by a pharmacist at the last visit within the intervention period (100 days after start of treatment); in addition, every week during the intervention period patients are asked how many days they took the study medication in the last week (0–7). No other measures (e.g., of blood or urine) will be used to assess dose compliance. We have not pre-defined a fixed limit for compliance since this was not defined in the previous study and 94–5% of the patients in that study consumed 95–100% of the tablets [18]. We will perform primary intention-to-treat (ITT) analyses of all patients regardless of their compliance. We may assess the effect of compliance on outcome in an exploratory analysis.

To assess patients’ blinding to treatment allocation, patients are asked post treatment (100 days after start of treatment) and at 1-year follow-up to report which study medicine they think that they received (antibiotics/placebo/unsure). The effect of their reports on outcome will be examined in explorative analysis.

### Data integrity

All clinician-reported and patient-reported data are captured electronically using Viedoc™ (Viedoc™, Pharma Consulting Group, Uppsala, Sweden), a web-based data capture system, compliant with all relevant regulations. The patients enter data in the ViedocMe application after logging on with their own unique username and password. An SMS is sent to the patients as a reminder 2 days before the deadline for completing the patient-reported data. Every week, local study coordinators at each study site control for data completeness for patients included at their own clinic. In the case of missing data, clinicians and/or patients are contacted in order to discover the cause.

### Sample size

The study is designed to assess the treatment effect in the total sample as well as separately in each MC type group (I/II). In each MC type group, the study is designed to detect (*β* = 0.1, two-sided *α* = 0.05) a mean difference of 4 (standard deviation (SD) 5) in the RMDQ score between the two treatment groups (amoxicillin or placebo) at 1-year follow-up. The SD of 5 is within the upper range of reported SDs for the RMDQ in patients with persistent LBP [40, 45–49]. In each MC type group, these assumptions result in a sample size of 66 (33 in each treatment group) (http://www.openepi.com/SampleSize/SSMean.htm). To enable separate analyses in each of the two MC type groups, we need 132 patients. (In the total sample, this provides 90% power at two-sided *α* = 0.05 to detect a mean difference of 2.8 (SD 5) in the RMDQ score between the two treatment groups). Presuming 20% dropouts (26 patients), we will include 80 MC type I patients and 80 MC type II patients. We plan to continue inclusion until 80 patients are included in the MC type group that is slowest to recruit, implying inclusion of at least 80 patients in the other MC type group and at least 160 patients in total.

### Data analysis

The pre-specified hypotheses have a prioritised order (Table 1), reducing multiple testing problems for the first hypothesis. A statistician blinded to treatment group will perform ITT analyses of the treatment effect on the primary outcome (RMDQ score at 1-year follow-up) in the total sample and in each MC type group (hypotheses A (main objective), B and C (secondary objectives), Table 1), using analysis of covariance (ANCOVA) adjusted for baseline RMDQ score. The significance level will be 0.05 in these analyses. Treatment effect evaluated by the secondary outcomes ODI and LBP-intensity scores at 1-year follow-up (hypotheses D and E, Table 1) will also be analysed using ITT ANCOVA adjusted for baseline score. In all analyses, we will report the between-group mean differences with 95% confidence intervals (CIs) and *p* values.

Secondary ITT analyses of treatment effect are (1) linear mixed-effects models (LME) for repeated measures of RMDQ (at baseline and at 3, 6, 9 and 12 months) and LBP intensity (at baseline, weekly during the treatment period, and at 3, 6, 9 and 12 months) and (2) responder analyses, comparing proportions of patients with > 75%, > 50% and > 30% reduction in RMDQ score from baseline to 1-year follow-up between treatment groups (chi-square tests) and reporting number needed to treat (NNT) with 95% CI.

For hypotheses F and G (STIR signal of MCs), the primary analysis will be multiple regression analysis. For hypothesis H (quality of life), ITT ANCOVA adjusted for baseline score will be used (Table 1). Additionally, we will consider exploratory per-protocol analyses for all hypotheses.

Missing 1-year data on RMDQ, ODI, LBP intensity and EuroQoL-5D, respectively, in the ITT ANCOVA analyses and in the responder analyses will be replaced using multiple imputation methods.

The research team will not perform interim analyses. An independent Data Monitoring Committee, blinded to treatment arm, will analyse the primary outcome measure (RMDQ) at 1-year follow-up in the first 80 included patients. They may stop the trial if they detect a mean difference of > 7.0 in 1-year RMDQ score between the two treatment arms, adjusted for baseline RMDQ score.

### Cost-effectiveness

The cost-effectiveness analysis will compare the potential effect of the treatment by using the primary outcome RMDQ and the EuroQoL-5D as measures of effectiveness. Costs of the study treatment (direct costs) will be estimated using a bottom-up approach. Costs to the healthcare system incurred due to LBP (indirect costs) will be estimated based on data recorded in a monthly cost diary. The diary will include number of visits to a general practitioner, physical or manual therapist, medical specialists, social worker, and alternative therapist; number of days of hospitalisation and/or rehabilitation; use of medication (both prescription and over-the-counter medication), and self-reported days of sick leave. The costs of work absenteeism will be estimated as the number of days absent from work multiplied by the average wage rate.

### MRI studies

MRI of the lumbar spine is performed at baseline and 1-year follow-up and includes T1- and T2-weighted fast spin-echo images (to assess presence and type of MCs) and STIR images (to provide fat saturation and test hypotheses F and G, Table 1). The MRIs also include T1-and T2-weighted fat-water separation images, diffusion-weighted images, and T1-weighted, fat-saturated, contrast-enhanced images.

We will determine the reliability (observer agreement) of different MC characteristics by different MRI methods (kappa statistics and Bland-Altman plots), and explore the relationship of different MC characteristics to each other and to clinical variables and outcomes (multiple regression analyses). We will also compare change in MC characteristics from baseline to 1-year follow-up between treatment groups.

### Biological and molecular studies

To identify and characterise novel biomarkers for MCs, we will assess underlying biological and molecular mechanisms. Epigenetic patterns, gene and protein expression and genetic variation will be mapped out, evaluated in the two treatment groups and for the two MC types at different time points, and correlated to clinical data. We will also investigate the impact of amoxicillin on the faecal flora and the emergence of resistant bacteria and resistance genes.

## Discussion

This article presents the design and rationale for a double-blind RCT comparing the effect of amoxicillin and placebo for patients with chronic LBP and MCs type I or II at the level of a previously herniated disc. A previous Danish study found an effect of amoxicillin clavulanate in patients with type I MCs [18]. If our findings differ, this may contribute to the prevention of inappropriate clinical use of antibiotics in a large patient population.

Several choices made when designing this study need discussion. First, the requirement of a prior disc herniation was due to the fact that bacterial investigations have almost solely been performed in herniated disc material [28]. In a biopsy study no viable bacteria were found in vertebrae affected with large MC type I changes [50]. We also wished to be able to report conclusive results and avoid overlooking any treatment effect in patients with disc herniation, since the Danish trial only included and found treatment effect in such patients [18]. Including patients without disc herniation would thus have required a doubled sample size (equally large groups with and without herniation) and financial support. We could have abandoned the separate investigation of treatment effect in each MC type group (which also required a doubled sample size), but we prioritised this investigation.

Second, and partly in order to re-assess findings in the Danish trial, we chose treatment with amoxicillin in a similar dose (750 mg three times a day, average of their high- and low-doses) and for the same period of time (100 days).

Regarding patient inclusion, since MC type is not a fixed characteristic (but may depend on MRI magnet strength), we decided to continue inclusion of both MC type groups unchanged until the MC type group that is slowest to recruit contains 80 patients (and the other MC type group contains at least 80 patients). Otherwise, we would also have had to exclude and potentially disappoint many eligible patients after their baseline MRI had shown MCs of a type no longer needed in the study. Such patients could potentially demotivate other eligible patients from participating in the study, which could reduce the representativeness of our study sample. Excluding patients earlier (prior to baseline MRI) based on MC type on the non-standardised clinical MRI would have induced heterogeneous patient selection.

For the sample size calculations we considered a between-group difference of 4 points on the RMDQ as the smallest clinically relevant difference. In the Danish study the difference between the amoxicillin clavulanate group and the placebo group at the 1-year follow-up was about 7 RMDQ points. In individual patients we consider a 30% improvement as the smallest clinically important reduction. However, taking the high dose and long duration of the antibiotic treatment into account, the size of the between-group differences, or individual improvement that is large enough to be considered clinically relevant, is not settled and is an issue open for discussion. In addition, since our study is designed to detect the smallest clinically relevant difference separately in each Modic type group, it may detect a smaller and irrelevant difference in the total sample. However, statistically significant differences of < 4 RMDQ points are not clinically relevant and will not be used as a basis for recommending antibiotic treatment in the studied patient groups. In addition, proper use of antibiotics must be based on interpretation of all relevant data on benefits and harms from all relevant studies.

### Trial status

Participant recruitment was initiated on 6 June 2015 and was ongoing at the time that this paper was submitted (16 March 2017). The last patient was recruited on 1 September 2017 (treatment started at 29 September 2017). A total of 180 patients are included. Data collection is expected to be complete by September 2018. (Additional file 1).

## Additional file

Additional file 1: SPIRIT 2013 Checklist. (DOC 121 kb)

## Abbreviations

BMI: Body Mass Index
CRF: Case Report Form
ITT: Intention-to-treat
LBP: Low back pain
LME: Linear mixed-effects models
MC: Modic change
MRI: Magnetic resonance imaging
NNT: Number needed to treat
NRS: Numerical Rating Scale
ODI: Oswestry Disability Index
*P. acnes*: *Proprionibacterium acnes* bacteria
RCT: Randomised controlled trial
RMDQ: Roland Morris Disability Questionnaire
SD: Standard deviation
SLV: Norwegian Medicines Agency
STIR: Short tau inversion recovery
